# The C allele of *JAK2 *rs4495487 is an additional candidate locus that contributes to myeloproliferative neoplasm predisposition in the Japanese population

**DOI:** 10.1186/1471-2350-13-6

**Published:** 2012-01-17

**Authors:** Junko H Ohyashiki, Masayuki Yoneta, Hisashi Hisatomi, Tamiko Iwabuchi, Tomohiro Umezu, Kazuma Ohyashiki

**Affiliations:** 1 Department of Molecular Oncology, Institute of Medical Science, Tokyo Medical University, Tokyo, Japan; 2 Department of Materials and Life Science, Seikei University, Tokyo, Japan; 3 First Department of Internal Medicine, Tokyo Medical University, Tokyo, Japan; 4 Department of Molecular Science, Tokyo Medical University, Tokyo, Japan

**Keywords:** *JAK2 *V617F, SNP, myeloproliferative neoplasms

## Abstract

**Background:**

Polycythemia vera (PV), essential thrombocythemia (ET), and primary myelofibrosis (PMF) are myeloproliferative neoplasms (MPNs) characterized in most cases by a unique somatic mutation, *JAK2 *V617F. Recent studies revealed that *JAK2 *V617F occurs more frequently in a specific *JAK2 *haplotype, named *JAK2 *46/1 or GGCC haplotype, which is tagged by rs10974944 (C/G) and/or rs12343867 (T/C). This study examined the impact of single nucleotide polymorphisms (SNPs) of the *JAK2 *locus on MPNs in a Japanese population.

**Methods:**

We sequenced 24 *JAK2 *SNPs in Japanese patients with PV. We then genotyped 138 MPN patients (33 PV, 96 ET, and 9 PMF) with known *JAK2 *mutational status and 107 controls for a novel SNP, in addition to two SNPs known to be part of the 46/1 haplotype (rs10974944 and rs12343867). Associations with risk of MPN were estimated by odds ratios and their 95% confidence intervals using logistic regression.

**Results:**

A novel locus, rs4495487 (T/C), with a mutated T allele was significantly associated with PV. Similar to rs10974944 and rs12343867, rs4495487 in the *JAK2 *locus is significantly associated with *JAK2*-positive MPN. Based on the results of SNP analysis of the three *JAK2 *locus, we defined the "GCC genotype" as having at least one minor allele in each SNP (G allele in rs10974944, C allele in rs4495487, and C allele in rs12343867). The GCC genotype was associated with increased risk of both *JAK2 *V617F-positive and *JAK2 *V617F-negative MPN. In ET patients, leukocyte count and hemoglobin were significantly associated with *JAK2 *V617F, rather than the GCC genotype. In contrast, none of the *JAK2 *V617F-negative ET patients without the GCC genotype had thrombosis, and splenomegaly was frequently seen in this subset of ET patients. PV patients without the GCC genotype were significantly associated with high platelet count.

**Conclusions:**

Our results indicate that the C allele of *JAK2 *rs4495487, in addition to the 46/1 haplotype, contributes significantly to the occurrence of *JAK2 *V617F-positive and *JAK2 *V617F-negative MPNs in the Japanese population. Because lack of the GCC genotype represents a distinct clinical-hematological subset of MPN, analyzing *JAK2 *SNPs and quantifying *JAK2 *V617F mutations will provide further insights into the molecular pathogenesis of MPN.

## Background

Myeloproliferative neoplasms (MPNs) represent a heterogeneous group of hematological malignancies characterized by clonal hematopoiesis and an increased number of mostly peripheral blood elements of myeloid origin [1]. The classic Philadelphia-chromosome negative MPNs encompass three distinct diseases, namely polycythemia vera (PV), essential thrombocythemia (ET), and primary myelofibrosis (PMF) [2-5]. Identification of the V617F mutation of the *JAK2 *gene (*JAK2 *V617F) led to an important breakthrough in the understanding of MPN disease pathogenesis [2-5]. The *JAK2 *V617F mutation is present in the majority of PV patients, and about 50% of patients with ET and PMF are affected [2-5]. Because this somatic mutation is highly specific to MPNs, it has been designated as a major diagnosis criterion for PV, ET, and PMF according to the latest World Health Organization classification of MPNs [6].

Recent investigations revealed that somatic acquisition of genetic aberrations is one pathogenic mechanism, but inherited genetic factors also play an important role in the development of MPN. Several independent groups reported that a particular *JAK2 *haplotype, designated 46/1 or GGCC, is strongly associated with the development [7-9], or with MPN development, regardless of the *JAK2 *mutational status [10,11]. Olcaydu et al. [12] performed *JAK2 *haplotype analysis in familial MPNs, and they concluded that even if *JAK*2 46/1 is related to the development of MPN independent of V617F status, it has to be regarded as only one of the genetic factors involved in the development of MPN. Moreover, Jones et al. [13] found correlations in *JAK*2 wild-type MPN between *JAK2 *46/1 and both MPL exon 10 and *JAK2 *exon 12.

In the present study, we attempted to find novel single nucleotide polymorphisms (SNPs) of the *JAK2 *locus in a Japanese population. We then examined whether *JAK2 *SNPs are indeed associated with a predisposition to MPNs, especially in *JAK2 *V617F-positive MPNs.

## Methods

### Patients

In the current study conducted at the Tokyo Medical University Hospital, 138 constitutive Japanese MPN patients aged 30-87 years with known *JAK2 *V617F status were included: 33 patients with *JAK2 *V617F-positive PV, 57 patients with *JAK2 *V617F-positive ET, 39 patients with *JAK2 *V617F-negative ET, and 9 patients with PMF. The patients experienced no familial MPNs. We revised their classification at diagnosis according to the latest World Health Organization classification of MPNs. As controls, 107 healthy volunteers aged 24-86 years from the same demographic area in Japan were used. The *JAK2 *V617F mutation detection system used was reported elsewhere [14], and the *JAK2 *V617F mutational status was categorized according to the allele burden of mutated T allele. This study was approved by the institutional review board of Tokyo Medical University (no. 975). Written informed consent according to the Declaration of Helsinki was obtained from all patients prior to collection of the specimens.

### PCR direct sequencing of the *JAK2 *locus

**Figure 1:**
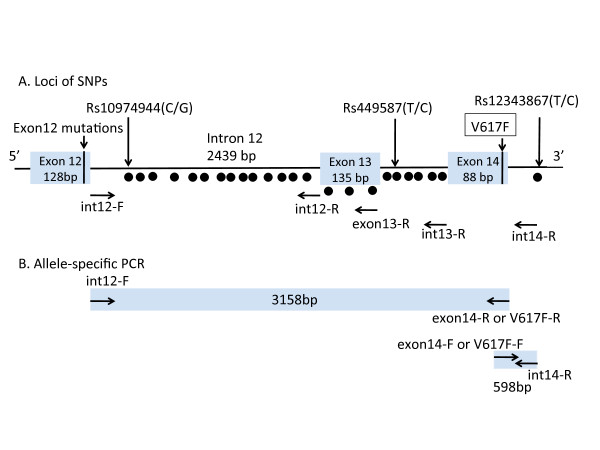
**Schematic representation of the *JAK2 *locus**. (A) Loci of SNPs are shown as black dots. Two SNPs included in the 46/1 haplotype are shown at the right end (rs12343867) and left end (rs10974944). A new candidate SNP is shown in the middle of those, near exon 13. Primers used for sequencing, a forward primer (int-12F) and four reverse primers (int-12-R, exon13-R, int-13R, and int-14R), are shown. (B) Location of primers used for allele-specific PCR. The larger fragment (3158 bp) was amplified using a forward primer (int-12F) and two sets of reverse primers (exon14-R or V617F-R) that can discriminate two alleles with or without *JAK2 *V617F. The smaller fragment was amplified using two forward primers (exon 14-F or V617F-F) and one reverse primer (int-14R). Detailed information on primers is given in Additional File 1.

Genomic DNA was obtained from whole blood using an automated system (Qiagen). To identify novel SNPs in the *JAK2 *locus in this Japanese population, primer sets for the amplification of *JAK2 *were designed according to GenBank AL161450 (Figure 1A; Additional file 1A). PCR conditions were 94°C for 30 s, 58°C for 30 s, and 72°C for 3 min for 36 cycles using High Fidelity^PLUS ^PCR System dNTPack (Roche Diagnostics, Mannheim, Germany). The PCR products were purified by a High Pure PCR Product Purification Kit (Roche Molecular Biochemical Diagnostics, Indianapolis, IN, USA) and sequenced using a BigDye Terminator v3.1 Cycle Sequencing Kit (Applied Biosystems, Foster City, CA, USA) with an Applied Biosystems 3130 Genetic Analyzer. The obtained sequences were compared with the *JAK2 *sequence.

### Allele-specific PCR analysis

To determine whether minor alleles of *JAK2 *SNPs favor the *cis *acquisition of *JAK2 *V617F, we performed allele-specific analysis of six SNPs in patients with PV. The sequence *JAK2 *nt51936-nt55084 (3158 bp) was amplified using forward primer int12-F and reverse primer V617F-R or exon14-R. The primer set for the amplification of *JAK2 *nt55038-nt55636 (598 bp) was forward primer V617F-F or exon14-F and reverse primer int14-R. Both primers V617F-R and V617F-F could amplify the T allele of *JAK2 *V617F (Figure 1B; Additional file 1A). The PCR products were purified and sequenced as described above.

### Genotyping

Allele-specific PCR was performed with a common forward primer and two allele-specific reverse primers (Additional file 1B) using High Fidelity^PLUS ^PCR System dNTPack (Roche Diagnostics) and SYBR Green I (Lonza, Rockland, ME, USA). The PCR conditions were 94°C for 30 s, 58°C for 30 s, and 72°C for 3 min for 40 cycles using an iCycler iQ Real-time PCR System (Bio-Rad Laboratories, Hercules, CA, USA). To avoid nonspecific PCR products, melting analysis was performed by denaturing at 95°C for 1 min and cooling to 55°C for 1 min followed by heating at the rate of 0.5°C/10 s from 55 to 95°C.

### Statistical analysis

GraphPad Prism 5.0 software (GraphPad Software Inc., San Diego, CA, USA) was used for statistical analysis. Associations with risk of MPNs were estimated by odds ratios (ORs) and their 95% confidence intervals (95% CIs) using logistic regression. A Mann-Whitney *U*-test was used to determine the statistical significance of differences between the control and test groups. *P*-values less than 0.05 were considered to indicate statistically significant differences. We also performed multivariate analysis using College analysis software (version 4.5, Fukuyama Heisei University, Fukuyama, Japan) to exclude possible false correlation between genotype and clinical manifestations.

## Results

### Minor allele frequency of SNPs from the *JAK2 *locus in Japanese PV patients

We first screened for 24 SNPs of the *JAK2 *locus around exons 12 to 14 in 28 Japanese patients with *JAK2 *V617F-positive PV from whom we obtained sufficient DNA for this analysis (Figure 1A). Among them, minor allele frequencies in six SNPs (rs10974944, rs12686652, rs12335546, rs4495487, rs1028730, and rs12343867) were significantly higher in PV patients than in healthy volunteers (Table 1). There were no significant differences in age or sex between the PV population and healthy controls (data not shown). Minor allele frequency was estimated by total cases that had at least one minor allele (heterozygous) or two minor alleles (homozygous). The *JAK2 *SNP rs4495487, which has not been reported previously in Caucasian populations, showed the highest OR (13.8, 95% CI: 3.79-50.21) among the six SNPs.

**Table 1:** Minor allele frequency of SNPs from the *JAK2 *locus in PV

No.	SNP	Minor alleles in PV (*n *= 28)			Minor alleles in control (*n *= 28)			*P *value* (Chi square test)	Odds ratio (95% CI)
				
		No	Yes (homo/hetero)	%	No	Yes (homo/hetero)	%		
1	rs10974944 (c→g)	7	21 (15/6)	75	17	11 (3/8)	39.2	0.0069	4.64 (1.49-14.55)
12	rs12686652 (c→g)	6	22 (21/1)	78.6	14	14 (5/9)	50	0.0257	3.67 (1.14-11.79)
13	rs12335546(c→t)	6	22 (21/1)	78.6	19	9 (1/8)	32.1	0.0005	7.74 (2.33-25.75)
19	rs4495487 (t→c)	7	21 (21/0)	75	23	5 (3/2)	17.8	< 0.0001	13.8 (3.79-50.21)
22	rs1028730 (g→a)	6	22 (21/1)	78.5	19	9 (2/7)	32.1	0.0005	7.74 (2.33-25.75)
24	rs12343867(t→c)	7	21 (21/0)	75	17	11 (4/9)	39.3	0.0069	4.64 (1.48-14.55)

* *P *values were calculated by the cases having minor alleles (homozygous and heterozygous) and cases without minor alleles.

To determine whether minor alleles of *JAK2 *SNPs favor the *cis *acquisition of *JAK2 *V617F, we next sequenced six SNPs using allele-specific primers (Additional file 1A). In accordance with a previous report [8], in the genotype with minor alleles in all six SNPs, the T allele was more frequently observed in *JAK2 *V617F than the G allele in normal controls; the OR was 7.74 (95% CI: 2.32-25.75) (Additional file 2).

### *JAK2 *SNP distribution in MPN patients and controls

We genotyped 138 MPN patients with known *JAK2 *mutational status and 107 controls for *JAK2 *SNP rs449587 in addition to two SNPs that are known to be part of the 46/1 haplotype (rs10974944 and rs12343867). Overall, 95 MPN patients (68.8%) were *JAK2 *V617F positive. When analyzing each MPN entity separately, we found that 33 PV patients (100%), 57 ET patients (63.3%), and 5 PMF patients (55.5%) were *JAK2 *V617F positive. The distribution of *JAK2 *SNPs (rs10974944, rs4495487, and rs12343867) is listed in Table 2. Allelic variation of three *JAK2 *SNPs was strongly associated with *JAK2*-positive MPN (all patients with PV, 57 patients with ET, and 5 patients with PMF) and much less strongly associated with *JAK2*-negative MPN (39 patients with ET and 4 patients with PMF). It is notable that allelic variation of the *JAK2 *SNP rs4495487 showed the highest OR in each population. The *JAK2 *SNP variation in 9 PMF patients is listed in Additional file 3. Because allelic variation of rs4495487 showed the highest OR in each patient population, we arbitrarily named genetic variation as the "GCC genotype," which has at least one minor allele in each of the three SNPs (G allele in rs10974944, C allele in rs4495487, and C allele in rs12343867); patients having the 46/1 haplotype and C allele of rs4495487. The GCC genotype is strongly associated with *JAK2 *V617F-positive MPNs (OR: 3.07; 95% CI: 1.73-5.46) and modestly associated with *JAK2 *V617F-negative MPN (OR: 2.26, 95% CI: 1.01-4.7) compared to normal controls (Table 3). The occurrence of the GCC genotype, however, did not depend on *JAK2 *V671F allele burden (Table 4).

**Table 2:** Genotype-specific association of SNPs from the *JAK2 *locus in MPN

Case population	Control population	SNP	*P*	**Genotype**^¶^			Odds ratio (95% CI)	
				Major	Hetero	Homo	Major vs Hetero	Major vs Homo
JAK2 V617F-positive PV	Healthy volunteers	rs10974944	0.0126*	CC	CG	GG	2.75 (1.06-7.14)*	4.24 (1.51-11.92)*
(*n *= 33)	Japanese (*n *= 107)	rs4495487	< 0.0001*	TT	TC	CC	4.26 (1.56-11.61)*	11.31 (3.60-35.57)*
		rs12343867	0.0032*	TT	TC	CC	2.26 (0.88-5.77)	5.683 (1.98-16.35)*
JAK2 V617F-positive ET	Healthy volunteers	rs10974944	0.0371*	CC	CG	GG	2.42 (1.20-4.92)*	1.10 (0.40-3.02)
(*n *= 57)	Japanese (*n *= 107)	rs4495487	0.0009*	TT	TC	CC	3.97 (1.86-8.49)*	4.00 (1.44-11.14)*
		rs12343867	0.0013*	TT	TC	CC	3.52 (1.68-7.38)*	3.55 (1.33-9.52)*
JAK2 V617F-negative ET	Healthy volunteers	rs10974944	0.068	CC	CG	GG	2.58 (1.14-5.84)*	1.49 (0.50-4.46)
(*n *= 39)	Japanese (*n *= 107)	rs4495487	0.0097*	TT	TC	CC	3.28 (1.45-7.43)*	3.05 (0.95-9.58)
		rs12343867	0.112	TT	TC	CC	2.26 (1.01-5.04)*	2.08 (0.67-6.40)
JAK2 V617F-positive MPN	Healthy volunteers	rs10974944	0.0138*	CC	CG	GG	2.42 (1.29-4.55)*	2.24 (1.03-4.88)*
(PV, ET, and PMF, *n *= 95)	Japanese (*n *= 107)	rs4495487	< 0.0001*	TT	TC	CC	4.08 (2.13-7.84)*	6.88 (2.88-16.41)*
		rs12343867	0.0002*	TT	TC	CC	2.89 (1.53-5.45)*	5.54 (2.35-13.06)*
JAK2 V617F-negative MPN	Healthy volunteers	rs10974944	0.1961	CC	CG	GG	2.01 (0.92-4.37)	1.14 (0.39-3.37)
(PV, ET, and PMF, *n *= 43)	Japanese (n = 107)	rs4495487	0.024*	TT	TC	CC	2.79 (1.28-6.08)*	2.48 (0.78-7.80)
		rs12343867	0.3069	TT	TC	CC	1.78 (0.82-3.84)	1.63 (0.58-4.94)
JAK2 V617F-positive MPN	JAK2 V617F-negative MPN	rs10974944	0.5493	CC	CG	GG	1.21 (0.55-2.66)	1.82 (0.70-5.35)
(PV, ET, and PMF, *n *= 95)	(PV, ET and PMF, *n *= 43)	rs4495487	0.1762	TT	TC	CC	1.46 (0.66-3.31)	2.78 (0.93-6.29)
		rs12343867	0.1572	TT	TC	CC	1.62 (0.72-3.61)	2.71 (0.93-7.90)

*P *based on chi-square test. Asterisks indicate statistically significant values.

^¶^Genotype was divided into three groups. Major: no existence of minor allele; Hetero: existence of one minor allele; Homo: existence of two minor alleles.

**Table 3:** Summary of the JAK2 genotype in MPN patients

	GCC genotype	non-GCC genotype	*P *(Chi-square test)	Odds ratio	95% CI
				
Case Population	46/1 haplotype rs4495987(+)	non-46/1 haplotype rs4495987(-)			
JAK2V617F-positive PV (*n *= 33)	22	11	0.0023	3.63	1.59-8.29
JAK2V617F-positive ET (*n *= 57)	35	22	0.0043	2.72	1.40-5.32
JAK2V617F-negative ET (*n *= 39)	24	15	0.0076	2.91	1.36-6.19
JAK2V617F-positive MPN (*n *= 95)	60	35	0.0001	3.07	1.73-5.46
JAK2V617F-negative MPN (*n *= 43)	24	19	0.0289	2.26	1.01-4.70
Control: non-MPN (*n *= 107)	38	69			

**Table 4:** *JAK2 *V617F allele burden and GCC genotype

Category	Genotype		*JAK2 *V617F allele burden			*P *value
			
		negative	< 20%	20-80%	> 80%	
PV	GCC (-)	0	0	7	3	
	GCC (+)	0	0	18	5	
ET	GCC (-)	17	8	14	1	0.4198
	GCC (+)	22	14	15	5	
PMF	GCC (-)	4	1	1	0	
	GCC (+)	0	0	2	1	
MPN	GCC (-)	21	8	22	4	0.6815
	GCC (+)	22	15	36	10	

### Clinical and hematological features, *JAK2 *V617F, and the GCC genotype

**Figure 2:**
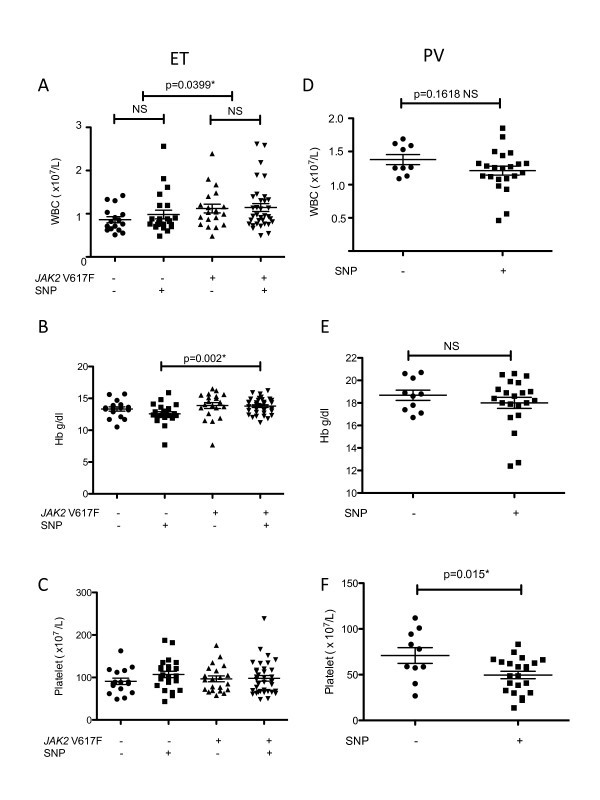
**Comparison of hematological features in ET and PV patients divided by *JAK2 *V617F mutational status and/or germline genetic variation (GCC genotype)**. (A) White blood cell (WBC) counts were significantly elevated in patients with *JAK2 *V617F-positive ET, regardless of GCC genotype (*p *= 0.0399). (B) Hemoglobin (Hb) levels were significantly elevated in ET patients having both *JAK2 *V617F and GCC genotype compared to those showing the GCC genotype but lacking *JAK2 *V617F (*p *= 0.002). (C) There was no significant difference in platelet count among ET groups. (D) Leukocyte count in PV patients. (E) Hemoglobin levels in PV patients. (F) Platelet count was significantly elevated in PV patients without the GCC genotype (*p *= 0.015).

We compared the clinical and hematological features of PV and ET patients with or without the GCC genotype. We subdivided ET patients into four groups: *JAK2 *V617F-negative ET patients with or without the GCC genotype and *JAK2 *V617F-positive ET patients with or without the GCC genotype (Table 5). None of the *JAK2 *V617F-negative ET patients without the GCC genotype had thrombosis (*p *= 0.0446), and splenomegaly was more frequently seen in this subset of ET patients (*p *= 0.0448), indicating that *JAK2 *V617F-negative ET without genetic variation shows a distinct clinical feature. White blood cell counts were significantly elevated in patients with *JAK2 *V617F-positive ET, regardless of GCC genotype status (*p *= 0.0399) (Figure 2A). Hemoglobin levels were significantly elevated in patients with both *JAK2 *V617F and the GCC genotype compared to those with the GCC genotype but lacking *JAK2 *V617F (*p *= 0.002) (Figure 2B). There was no significant difference in platelet counts among the four groups. These results indicate that the proliferative nature of MPNs, such as increased white blood cell and hemoglobin counts, may be linked to *JAK2 *V617F, regardless of *JAK2 *genetic variations.

**Table 5:** Demographic and clinical characteristics of ET patients

	*JAK2 *V617F negative		JAK2 V617F positive		
		
	GCC genotype		GCC genotype		*P *value
	No (*n *= 19)	Yes (*n *= 23)	No (*n *= 22)	Yes (*n *= 35)	
Age, mean (SD)	51.2 (18.3)	57.9 (17.5)	64.8 (14.9)	66.1 (16.2)	0.6466
Sex					
Female	10 (52.6%)	14 (60.9%)	8 (36.4%)	22 (62.9%)	0.2965
Male	9 (47.4%)	9 (30.1%)	14 (64.6%)	13 (37.1%)	
Splenomegaly	11/16 (68.8%)	4/22 (18.2%)	5/20 (25%)	4/33 (12.1%)	0.0448*
Thrombosis	0/11 (0%)	5/21 (23.8%)	6/17 (35.3%)	7/30 (23.3%)	0.0446*
Therapy requirement	10/16 (90%)	15/22 (68.2%)	7/21 (33.3%)	25/34 (73.5%)	0.7408
Cytogenetic abnormality	1/8(0%)	0/16 (0%)	2/9 (22.2%)	4/21(19%)	0.3618
MF evolution	0/16 (0%)	1/22 (4.5%)	0/15 (0%)	1/30 (3.3%)	0.3787
AML evolution	0/11 (0%)	1/22 (4.5%)	0/15 (0%)	0/30 (0%)	0.7343
Survival, mean (SD)	2036 (2267)	1883 (1643)	1706 (1847)	1717 (292)	0.6528

Statistical analysis was done among four groups.

*P *value is obtained from multivariate analysis.

Asterisks indicate statistically significant values.

Because all the PV patients in the current study were *JAK2 *V617F positive, we compared the clinical and hematological features between PV patients with or without the GCC genotype (Table 6). Although there were no significant differences in age, sex, clinical manifestations, or survival between the two groups, platelet count was significantly elevated in PV patients without the GCC genotype (*p *= 0.015) (Figure 2). These findings suggest that germline genetic variation also affects PV patients.

**Table 6:** Demographic and clinical characteristics of PV patients

	GCC genotype		
		
	No (*n *= 10)	Yes (*n *= 23)	*P *value
Age, mean (SD)	63.2 (12.4)	60.7 (14.0)	0.5779
Sex			
Female	4 (40%)	13 (56.7%)	0.4646
Male	6 (60%)	10 (43.5%)	
Splenomegaly	5/10 (50%)	12/23 (52.2%)	0.7413
Thrombosis	3/10 (30%)	4/20 (20%)	0.4786
Therapy requirement	9/10 (90%)	22/23 (95.7%)	0.2585
Cytogenetic abnormality	0/10 (0%)	3/23 (13%)	0.1781
MF evolution	3/10 (30%)	3/23 (13%)	0.0924
AML evolution	1/10 (10%)	3/23 (13%)	0.7614
Survival, median (SD)	3427 (2336)	2840 (2822)	0.7583

*P *value is obtained from multivariate analysis.

## Discussion

This is the first study to provide evidence of an association between somatic *JAK2 *V617F mutation and *JAK2 *SNPs in a Japanese population of MPN patients. We found a candidate SNP, rs4495487, that may contribute to MPN phenotype in this population. A contribution of this SNP has not been reported in Caucasian populations; however, because it is located between rs1097944 and rs12343867, rs4495487 might be included in the 46/1 haplotype. As in previous reports, we found a significant association between *JAK2 *SNPs and MPN phenotype in *JAK2 *V617F-positive MPNs [7-9] and in *JAK2 *V617F-negative MPNs [10,11].

Although the occurrence of *JAK2 *V617F greatly contributes to the diagnosis of MPNs, it remains unclear why this single genetic change represents at least three clinical phenotypes (i.e., PV, ET, and PMF). It also remains uncertain whether *JAK2 *V617F is the primary genetic change responsible for MPNs. Thus, the major obstacle to clarifying the molecular pathogenesis of MPNs is the substantial complexity of the genetic changes, including germline genetic variation of the *JAK2 *locus.

In the present study, we demonstrated an association between germline genetic variation in the *JAK2 *locus and MPN phenotype in a Japanese population. Although the clinical manifestation largely depends on *JAK2 *V617F mutation rather than SNPs in the *JAK2 *locus of ET patients, we noted that *JAK2 *V617F-negative ET without the GCC genotype showed a distinct clinical feature, suggesting an underlying genetic change that has not yet been identified. Tefferi et al. [11] demonstrated that nullizygosity for the *JAK2 *46/1 haplotype is associated with inferior survival. Taken together, these findings suggest that the lack of certain germline genetic variation may play an important role in the pathogenesis of MPNs. In a study by Trifa et al. [15], the 46/1 haplotype was associated with mutant allele burden > 50% in *JAK2 *V617F-positive MPN patients. However, we could not find any relationship between allele burden and germline genetic variations. Although we found an association between splenomegaly and *JAK2 *V617F-negative ET without the GCC genotype, a previous report by Vannucchi et al. [16] demonstrated *JAK2 *V617F mutation was related to larger spleen size in ET. In addition, we found no significant differences in platelet count among the ET groups, unlike previous reports [16,17]. These discrepancies could be related to differences in the size or ethnics of the analyzed patient cohorts. Therefore, larger studies of Japanese patients should be conducted to clarify the association between *JAK2 *V617 allele burden and *JAK2 *haplotype.

According to a recent report by Colaizzo et al. [18], in patients with splanchnic venous thrombosis, the *JAK2 *V617F mutation is frequently found in women and, when interacting with the 46/1 haplotype, it may represent a gender-related susceptibility allele for splanchnic venous thrombosis. In the current study, we found no relationships between sex or genotype and the occurrence of thrombosis. However, future research should clarify whether sex modulation of those genetic changes also occur in Asian populations. In the present study, none of the *JAK2 *V617F-negative ET patients without the GCC genotype had the complication of thrombosis. Smalberg et al. [19] recently demonstrated that the 46/1 haplotype is associated with the development of *JAK2 *V617F-positive splenic vein thrombosis (SVT), but the existence of *JAK2 *V617F-negative SVT patients also indicates an important role for the 46/1 haplotype in the etiology and diagnosis of SVT-related MPNs. In contrast, Kouroupi et al. [20] showed that the 46/1 haplotype is not a susceptibility locus for the development of SVT. Thus, further exploration is required to clarify whether the *JAK2 *germline variation is a risk factor of thrombosis. It is notable that platelet count was significantly higher in PV patients without the GCC genotype. Although this is partially due to the relative iron deficiency during the expansion of the PV clone, these findings suggest that a lack of germline genetic variation (i.e., nullizygosity) may be linked to disease severity.

There is mounting evidence of genetic causes of MPN initiation and progression besides *JAK2 *and *MPL*, which define the MPN phenotype [21-28]. Deletion or mutation of *TET2*, associated with deletion and UPDs of chromosome 4q, have been reported in 10-12% of MPNs with or without *JAK2 *V617F [23,28,29]. Mutation of *ASXL1 *in CD34-positive purified cells strongly suggests that this is an early genetic event closely related to epigenetic status [24]. Ernst et al. [25] reported that *EZH2*, which encodes the catalytic subunit of the polycomb repressive complex (PRC2), is mutated in a subset of MPNs; *EZH2 *also influences stem cell renewal by epigenetic repression of genes involved in cell fate decisions. In contrast, mutation of *IDH *is frequently found in blast-phase MPNs [26]. Therefore, investigation of other oncogenic mutations in MPN patients and their associations with germline gene variants might help to reveal the mechanism underlying the relationship between haplotype variants and somatic mutability.

## Conclusions

In conclusion, we demonstrated that the C allele of the *JAK2 *rs4495487 is an additional candidate locus that contributes to MPN predisposition in the Japanese population. Although the number of patients analyzed is too small to draw a definitive conclusion, our results provide novel insights into the molecular pathogenesis of MPNs. To clarify the pathophysiology of MPNs, it will be necessary to analyze *JAK2 *SNPs as a MPN predisposition, quantify *JAK2 *V617F mutations as a hallmark of MPN phenotype, and identify other germline variants and somatic mutations, including TET2, in a large number of patients.

## Competing interests

The authors declare that they have no competing interests.

## Authors' contributions

JHO participated in the design and interpretation of the analysis, statistical analysis, and writing of the article. HH planned and coordinated the research. KO, TI, and UT collected samples from patients, and MY performed DNA sequencing and PCR analysis. KO helped to write the article. All authors read and approved the final manuscript.

## Pre-publication history

The pre-publication history for this paper can be accessed here:

http://www.biomedcentral.com/1471-2350/13/6/prepub

## Supplementary Material

Additional file 1 **Primers used in this study**. (A) Primers used for PCR-direct sequencing. Primers 1, 7, 8, 9, and 10 were also used for allele-specific PCR to discriminate *JAK2 *V617F-positive and -negative alleles. (B) Primers used for SNP analysis in 138 MPN patients and 107 healthy controls. Click here for file

Additional file 2 **Six SNPs of the *JAK2 *locus in PV patients and normal controls**. Six SNPs were sequenced using allele-specific primers (Additional File 1A). In normal controls, detected SNPs were located in the allele without *JAK2 *V617F mutation. In PV patients, detected SNPs were located in the mutated T allele of *JAK2*. The genotype that had minor alleles in all six SNPs was designated as the GGTCAC genotype. This genotype is more frequently observed in T allele of *JAK2 *V617F (19/28, 67.9%) than G allele in normal controls (6/28, 21.4%); the odds ratio was 7.74 (95% CI: 2.32-25.75). There were no significant differences in age or sex between normal controls and patients with PV. Click here for file

Additional file 3 ***JAK2 *SNP distribution in 9 patients with PMF**. We could not statistically analyze the possible association between genotypes and clinical manifestations because of the small number of PMF patients in this single-institution study, however, results of genotypic analysis are shown in this table. Click here for file

## References

[B1] Jones AV Kreil S Zoi K Waghorn K Curtis C Zhang L Score J Seear R Chase AJ Grand FH White H Zoi C Loukopoulos D Terpos E Vervessou EC Schultheis B Emig M Ernst T Lengfelder E Hehlmann R Hochhaus A Oscier D Silver RT Reiter A Cross NC Widespread occurrence of the JAK2 V617F mutation in chronic myeloproliferative disorders Blood 2005 106 6 2162 2168 10.1182/blood-2005-03-1320 15920007

[B2] Kralovics R Passamonti F Buser AS Teo SS Tiedt R Passweg JR Tichelli A Cazzola M Skoda RC A gain-of-function mutation of JAK2 in myeloproliferative disorders N Engl J Med 2005 352 17 1779 1790 10.1056/NEJMoa051113 15858187

[B3] James C Ugo V Le Couedic JP Staerk J Delhommeau F Lacout C Garcon L Raslova H Berger R Bennaceur-Griscelli A Villeval JL Constantinescu SN Casadevall N Vainchenker W A unique clonal JAK2 mutation leading to constitutive signalling causes polycythaemia vera Nature 2005 434 7037 1144 1148 10.1038/nature03546 15793561

[B4] Levine RL Wadleigh M Cools J Ebert BL Wernig G Huntly BJ Boggon TJ Wlodarska I Clark JJ Moore S Adelsperger J Koo S Lee JC Gabriel S Mercher T D'Andrea A Fröhling S Döhner K Marynen P Vandenberghe P Mesa RA Tefferi A Griffin JD Eck MJ Sellers WR Meyerson M Golub TR Lee SJ Gilliland DG Activating mutation in the tyrosine kinase JAK2 in polycythemia vera, essential thrombocythemia, and myeloid metaplasia with myelofibrosis Cancer Cell 2005 7 4 387 397 10.1016/j.ccr.2005.03.023 15837627

[B5] Baxter EJ Scott LM Campbell PJ East C Fourouclas N Swanton S Vassiliou GS Bench AJ Boyd EM Curtin N Scott MA Erber WN Green AR Acquired mutation of the tyrosine kinase JAK2 in human myeloproliferative disorders Lancet 2005 365 9464 1054 1061 15781101 10.1016/S0140-6736(05)71142-9

[B6] Tefferi A Vardiman JW Classification and diagnosis of myeloproliferative neoplasms: the 2008 World Health Organization criteria and point-of-care diagnostic algorithms Leukemia 2008 22 1 14 22 10.1038/sj.leu.2404955 17882280

[B7] Jones AV Chase A Silver RT Oscier D Zoi K Wang YL Cario H Pahl HL Collins A Reiter A Grand F Cross NC JAK2 haplotype is a major risk factor for the development of myeloproliferative neoplasms Nat Genet 2009 41 4 446 449 10.1038/ng.334 19287382 PMC4120192

[B8] Kilpivaara O Mukherjee S Schram AM Wadleigh M Mullally A Ebert BL Bass A Marubayashi S Heguy A Garcia-Manero G Kantarjian H Offit K Stone RM Gilliland DG Klein RJ Levine RL A germline JAK2 SNP is associated with predisposition to the development of JAK2(V617F)-positive myeloproliferative neoplasms Nat Genet 2009 41 4 455 459 10.1038/ng.342 19287384 PMC3676425

[B9] Olcaydu D Harutyunyan A Jager R Berg T Gisslinger B Pabinger I Gisslinger H Kralovics R A common JAK2 haplotype confers susceptibility to myeloproliferative neoplasms Nat Genet 2009 41 4 450 454 10.1038/ng.341 19287385

[B10] Pardanani A Lasho TL Finke CM Gangat N Wolanskyj AP Hanson CA Tefferi A The JAK2 46/1 haplotype confers susceptibility to essential thrombocythemia regardless of JAK2V617F mutational status-clinical correlates in a study of 226 consecutive patients Leukemia 2010 24 1 110 114 10.1038/leu.2009.226 19847198

[B11] Tefferi A Lasho TL Patnaik MM Finke CM Hussein K Hogan WJ Elliott MA Litzow MR Hanson CA Pardanani A JAK2 germline genetic variation affects disease susceptibility in primary myelofibrosis regardless of V617F mutational status: nullizygosity for the JAK2 46/1 haplotype is associated with inferior survival Leukemia 2010 24 1 105 109 10.1038/leu.2009.225 19847199

[B12] Olcaydu D Rumi E Harutyunyan A Passamonti F Pietra D Pascutto C Berg T Jager R Hammond E Cazzola M Kralovics R The role of the JAK2 GGCC haplotype and the TET2 gene in familial myeloproliferative neoplasms Haematologica 2011 96 3 367 374 10.3324/haematol.2010.034488 21173100 PMC3046267

[B13] Jones AV Campbell PJ Beer PA Schnittger S Vannucchi AM Zoi K Percy MJ McMullin MF Scott LM Tapper W Silver RT Oscier D Harrison CN Grallert H Kisialiou A Strike P Chase AJ Green AR Cross NC The JAK2 46/1 haplotype predisposes to MPL-mutated myeloproliferative neoplasms Blood 2010 115 22 4517 4523 10.1182/blood-2009-08-236448 20304805 PMC3145114

[B14] Ohyashiki K Aota Y Akahane D Gotoh A Ohyashiki JH JAK2(V617F) mutational status as determined by semiquantitative sequence-specific primer-single molecule fluorescence detection assay is linked to clinical features in chronic myeloproliferative disorders Leukemia 2007 21 5 1097 1099 17315023 10.1038/sj.leu.2404604

[B15] Trifa AP Cucuianu A Petrov L Urian L Militaru MS Dima D Pop IV Popp RA The G allele of the JAK2 rs10974944 SNP, part of JAK2 46/1 haplotype, is strongly associated with JAK2 V617F-positive myeloproliferative neoplasms Ann Hematol 2010 89 10 979 983 10.1007/s00277-010-0960-y 20422415

[B16] Vannucchi AM Antonioli E Guglielmelli P Rambaldi A Barosi G Marchioli R Marfisi RM Finazzi G Guerini V Fabris F Randi ML De Stefano V Caberlon S Tafuri A Ruggeri M Specchia G Liso V Rossi E Pogliani E Gugliotta L Bosi A Barbui T Clinical profile of homozygous JAK2 617V > F mutation in patients with polycythemia vera or essential thrombocythemia Blood 2007 110 3 840 846 10.1182/blood-2006-12-064287 17379742

[B17] Patriarca A Pompetti F Malizia R Iuliani O Di Marzio I Spadano A Dragani A Is the absence of JAK2 mutation a risk factor for bleeding in essential thrombocythemia? An analysis of 106 patients Blood Transfus 2010 8 1 21 27 20104275 10.2450/2009.0004-09 PMC2809508

[B18] Colaizzo D Tiscia GL Bafunno V Amitrano L Vergura P Lupone MR Grandone E Guardascione MA Margaglione M Sex modulation of the occurrence of jak2 v617f mutation in patients with splanchnic venous thrombosis Thromb Res 2011 10.1016/j.thromres.2011.03.024 21497883

[B19] Smalberg JH Koehler E Darwish Murad S Plessier A Seijo S Trebicka J Primignani M de Maat MP Garcia-Pagan JC Valla DC Randi ML De Stefano V Caberlon S Tafuri A Ruggeri M Specchia G Liso V Rossi E Pogliani E Gugliotta L Bosi A Barbui T The JAK2 46/1 haplotype in Budd-Chiari syndrome and portal vein thrombosis Blood 2011 117 15 3968 3973 10.1182/blood-2010-11-319087 21364191

[B20] Kouroupi E Kiladjian JJ Chomienne C Dosquet C Bellucci S Valla D Cassinat B The JAK2 46/1 haplotype in splanchnic vein thrombosis Blood 2011 117 21 5777 5778 10.1182/blood-2011-03-343657 21617012

[B21] Pardanani AD Levine RL Lasho T Pikman Y Mesa RA Wadleigh M Steensma DP Elliott MA Wolanskyj AP Hogan WJ McClure RF Litzow MR Gilliland DG Tefferi A MPL515 mutations in myeloproliferative and other myeloid disorders: a study of 1182 patients Blood 2006 108 10 3472 3476 10.1182/blood-2006-04-018879 16868251

[B22] Steensma DP Caudill JS Pardanani A McClure RF Lasho TL Tefferi A MPL W515 and JAK2 V617 mutation analysis in patients with refractory anemia with ringed sideroblasts and an elevated platelet count Haematologica 2006 91 12 Suppl ECR57 17194663

[B23] Delhommeau F Dupont S Della Valle V James C Trannoy S Masse A Kosmider O Le Couedic JP Robert F Alberdi A Lécluse Y Plo I Dreyfus FJ Marzac C Casadevall N Lacombe C Romana SP Dessen P Soulier J Viguié F Fontenay M Vainchenker W Bernard OA Mutation in TET2 in myeloid cancers N Engl J Med 2009 360 22 2289 2301 10.1056/NEJMoa0810069 19474426

[B24] Carbuccia N Murati A Trouplin V Brecqueville M Adelaide J Rey J Vainchenker W Bernard OA Chaffanet M Vey N Birnbaum D Mozziconacci MJ Mutations of ASXL1 gene in myeloproliferative neoplasms Leukemia 2009 23 11 2183 2186 10.1038/leu.2009.141 19609284

[B25] Ernst T Chase AJ Score J Hidalgo-Curtis CE Bryant C Jones AV Waghorn K Zoi K Ross FM Reiter A Hochhaus A Drexler HG Duncombe A Cervantes F Oscier D Boultwood J Grand FH Cross NC Inactivating mutations of the histone methyltransferase gene EZH2 in myeloid disorders Nat genet 2010 42 8 722 726 10.1038/ng.621 20601953

[B26] Pardanani A Lasho TL Finke CM Mai M McClure RF Tefferi A IDH1 and IDH2 mutation analysis in chronic- and blast-phase myeloproliferative neoplasms Leukemia 2010 24 6 1146 1151 10.1038/leu.2010.77 20410924

[B27] Sanada M Suzuki T Shih LY Otsu M Kato M Yamazaki S Tamura A Honda H Sakata-Yanagimoto M Kumano K Oda H Yamagata T Takita J Gotoh N Nakazaki K Kawamata N Onodera M Nobuyoshi M Hayashi Y Harada H Kurokawa M Chiba S Mori H Ozawa K Omine M Hirai H Nakauchi H Koeffler HP Ogawa S Gain-of-function of mutated C-CBL tumour suppressor in myeloid neoplasms Nature 2009 460 7257 904 908 10.1038/nature08240 19620960

[B28] Tefferi A Pardanani A Lim KH Abdel-Wahab O Lasho TL Patel J Gangat N Finke CM Schwager S Mullally A Li CY Hanson CA Mesa R Bernard O Delhommeau F Vainchenker W Gilliland DG Levine RL TET2 mutations and their clinical correlates in polycythemia vera, essential thrombocythemia and myelofibrosis Leukemia 2009 23 5 905 911 10.1038/leu.2009.47 19262601 PMC4654629

[B29] Klampfl T Harutyunyan A Berg T Gisslinger B Schalling M Bagienski K Olcaydu D Passamonti F Rumi E Pietra D Jäger R Pieri L Guglielmelli P Iacobucci I Martinelli G Cazzola M Vannucchi AM Gisslinger H Kralovics R Genome integrity of myeloproliferative neoplasms in chronic phase and during disease progression Blood 2011 118 1 167 176 10.1182/blood-2011-01-331678 21531982

