# Supplementary material for: The C allele of JAK2 rs4495487 is an additional candidate locus that contributes to myeloproliferative neoplasm predisposition in the Japanese population
Source: BMC Med Genet. 2012 Jan 17;13:6. doi: 10.1186/1471-2350-13-6 (PMC3277458; doi:10.1186/1471-2350-13-6)
Supplement: Additional file 1 — Primers used in this study. (A) Primers used for PCR-direct sequencing. Primers 1, 7, 8, 9, and 10 were also used for allele-specific PCR to discriminate JAK2 V617F-positive and -negative alleles. (B) Primers used for SNP analysis in 138 MPN patients and 107 healthy controls. [file 1471-2350-13-6-S1.PDF]

Additional file 1

A. Primers used for PCR-direct sequencing

No.	Primer	Description	Sequence
1	int12-F*	Forward	5'-CAGAAATACTATATTTTCCAACTGAGTTTC-3'
2	int12-R	Reverse	5'-CCTGAATGGGCTATAATTGTCACTT-3'
3	exon13-R	Reverse	5'-AGCTGCACACATGAGTACGTTGT-3'
4	int13-R	Reverse	5'-CAGGAATCATGAATAATGCCAGTCAA-3'
6	*JAK2*-int14-R*	Reverse	5'-GTAAAAAATACAATATCTAAGAAGCAC-3'
7	*JAK2*V617F-mutated-R*	Reverse	5'-TTACTTACTCTCGTCTCCACAGBA-3'
8	*JAK2*-exon14-R *	Reverse	5'-TTACTCTCGTCTCCACAGAC-3'
9	*JAK2* V617F-mutated-F*	Forward	5'-TGGTTTTAAATTATGGAGTATGCT-3'
10	*JAK2*-exon14-F*	Forward	5'-TGGTTTTAAATTATGGAGTATGCG-3'

Asterisks indicate primers also used in JAK2V617F-positive or -negative allele-specific PCR

B. Primers used for SNP analysis

No.	Primer	Description	Sequences
1	rs10974944-G-F*	Forward	5'-GTGGTGAGGGTTGATGACG-3'
2	rs10974944-T-F	Forward	5'-TGGTGAGGGTTGATGATC-3'
3	rs10974944-R	Reverse	5'-TAGGTTAAGAGTATGTGGTTCCG-3'
4	rs4495487-F	Forward	5'-ATAATCCTTGTTTTCTGTCTC-3'
5	rs4495487-C-R *	Reverse	5'-TTAATTTTTTTAATCCGGTAG-3'
6	rs4495487-T-R	Reverse	5'-TTAATTTTTTTAATCCGGTGA-3'
7	rs12343867-F	Forward	5'-AACTATAATTTAACAGGAGT-3'
8	rs12343867-C-R *	Reverse	5'-TATATCTAGTATCATATCAACG-3'
9	rs12343867-T-R	Reverse	5'-TATATCTAGTATCATATCAACA-3'
