# Supplementary material for: The C allele of JAK2 rs4495487 is an additional candidate locus that contributes to myeloproliferative neoplasm predisposition in the Japanese population
Source: BMC Med Genet. 2012 Jan 17;13:6. doi: 10.1186/1471-2350-13-6 (PMC3277458; doi:10.1186/1471-2350-13-6)
Supplement: Additional file 2 — Six SNPs of the JAK2 locus in PV patients and normal controls. Six SNPs were sequenced using allele-specific primers (Additional File 1A). In normal controls, detected SNPs were located in the allele without JAK2 V617F mutation. In PV patients, detected SNPs were located in the mutated T allele of JAK2. The genotype that had minor alleles in all six SNPs was designated as the GGTCAC genotype. This genotype is more frequently observed in T allele of JAK2 V617F (19/28, 67.9%) than G allele in normal controls (6/28, 21.4%); the odds ratio was 7.74 (95% CI: 2.32-25.75). There were no significant differences in age or sex between normal controls and patients with PV. [file 1471-2350-13-6-S2.PDF]

Additional file 2

	rs10974944 c→g	rs12686652 c→g	rs12335546 c→t	rs4495487 t→c	rs10283730 g→a	rs12343867 t→c		rs10974944 c→g	rs12686652 c→g	rs12335546 c→t	rs4495487 t→c	rs10283730 g→a	rs12343867 t→c	JAK2 V617F
NC-1	G	G	T	C	A	C	PV-1	G	G	T	C	A	C	G/T
NC-2	G	G	T	C	A	C	PV-2	G	G	T	C	A	C	G/T
NC-3	G	G	T	C	A	C	PV-3	G	G	T	C	A	C	G/T
NC-4	G	G	C/T	C	A	C	PV-4	G	G	T	C	A	C	G/T
NC-5	G	G	T	T/C	A	C	PV-5	G	G	T	C	A	C	G/T
NC-6	G	G	T	C	A	C	PV-6	G	G	T	C	A	C	G/T
NC-7	G	G	T	T	A	C	PV-7	G	G	T	C	A	C	T/T
NC-8	G	G	T	T	A	C	PV-8	G	G	T	C	A	C	T/T
NC-9	G	G	T	T	A	C	PV-9	G	G	T	C	A	C	G/T
NC-10	G	G	T	T	A	C	PV-10	G	G	T	C	A	C	G/T
NC-11	G	C	T	T	A	C	PV-11	G	G	T	C	A	C	G/T
NC-12	G	G	C	T/C	G	T/C	PV-12	G	G	T	C	A	C	G/T
NC-13	G	G	C	T	G	T	PV-13	G	G	T	C	A	C	T/T
NC-14	C	G	C	T	G	T	PV-14	G	G	T	C	A	C	G/T
NC-15	C	C/G	C	T	G	T	PV-15	G	G	T	C	A	C	G/T
NC-16	C	C/G	C	T	G	T	PV-16	G	G	T	C	A	C	G/T
NC-17	C	C/G	C	T	G	T	PV-17	G	G	T	C	A	C	G/T
NC-18	C	C	C	T	G	T	PV-18	G	G	T	C	A	C	T/T
NC-19	C	C	C	T	G	T	PV-19	G	G	T	C	A	C	G/T
NC-20	C	C	C	T	G	T	PV-20	G	C	T	C	A	C	T/T
NC-21	C	C	C	T	G	T	PV-21	G	G	T	T	A	T	G/T
NC-22	C	C	C	T	G	T	PV-22	C	G	T	C	A	C	T/T
NC-23	C	C	C	T	G	T	PV-23	C	G	C	T	G	T	T/T
NC-24	C	C	C	T	G	T	PV-24	C	C	C	T	G	T	G/T
NC-25	C	C	C	T	G	T	PV-25	C	C	C	T	G	T	G/T
NC-26	C	C	C	T	G	T	PV-26	C	C	C	T	G	T	T/T
NC-27	C	C/G	C	T	G	T	PV-27	C	C	C	T	G	T	G/T
NC-28	C	C	C	T	G	T	PV-28	C	C	C	T	G	T	T/T

NC: normal control; PV: patients with *JAK2* V617F-positive PV

Patients exhibiting minor alleles at all five SNPs are marked in gray.
