# Supplementary material for: The C allele of JAK2 rs4495487 is an additional candidate locus that contributes to myeloproliferative neoplasm predisposition in the Japanese population
Source: BMC Med Genet. 2012 Jan 17;13:6. doi: 10.1186/1471-2350-13-6 (PMC3277458; doi:10.1186/1471-2350-13-6)
Supplement: Additional file 3 — JAK2 SNP distribution in 9 patients with PMF. We could not statistically analyze the possible association between genotypes and clinical manifestations because of the small number of PMF patients in this single-institution study, however, results of genotypic analysis are shown in this table. [file 1471-2350-13-6-S3.PDF]

Additional file 3. JAK2 SNP distribution in 9 patients with PMF

Patient no.	*JAK2* V617F	rs10974944 c>g	rs4495487 t>c	rs1234867 t>c
1	GG	CC	TT	TT
2	GG	CC	TT	TT
3	GT	CC	TT	TT
4	GT	CC	TT	TT
5	GG	CC	TT	TT
6	GT	CG	CT	CT
7	GG	CC	CT	TT
8	GT	GG	CC	CC
9	TT	GG	CC	CC
